# Caesarean Section for Orthopedic Indications

**DOI:** 10.3390/jcm12237336

**Published:** 2023-11-27

**Authors:** Maciej Ziętek, Paweł Ziętek, Daniel Kotrych, Małgorzata Szczuko

**Affiliations:** 1 Department of Perinatology, Obstetrics and Gynecology, Pomeranian Medical University in Szczecin, 71-010 Police, Poland; 2 Department of Orthopedics, Traumatology, Pomeranian Medical University in Szczecin, 71-252 Szczecin, Poland; pawel.zietek@pum.edu.pl (P.Z.); daniel.kotrych@pum.edu.pl (D.K.); 3 Department of Human Nutrition and Metabolomics, Pomeranian Medical University in Szczecin, 71-460 Szczecin, Poland; malgorzata.szczuko@pum.edu.pl

**Keywords:** caesarean section, pregnancy, orthopedic indications

## Abstract

Background: The increasing number of late complications described after cesarean sections is prompting a reexamination of the indications for them in pregnant women. The high percentage of pregnancies terminated by preventive cesarean section for non-obstetric reasons also largely involves orthopedic conditions. A challenge for obstetricians is pregnant patients with orthopedic conditions both before and during pregnancy. Pregnant women with a history of orthopedic surgery require special attention. The lack of consensus in this area, physicians’ fear of patients’ claims and the skewing of patients’ requests for surgical termination of pregnancy have prompted an analysis and systematization of existing knowledge in this field. Methods: References published up to 30 June 2023 in five databases Pubmed, Embase are included. Keywords have been checked for the following: pubic symphysis diastasis, lumbar disc herniation, past hip arthroplasty and fractures in the pelvic bones. In the described conditions complicating pregnancy, the mode of delivery was taken into account. Results: All included studies were screened and reviewed by at least two authors until an overall consensus of 50 articles was reached. Conclusions: Orthopedic indications for cesarean section in many cases should not be treated imperatively, since natural delivery after correct fusion of a pelvic fracture, implantation of a hip endoprosthesis or a limited dissection of the pubic symphysis is possible and is not associated with a higher risk of obstetric or orthopedic complications. Extra-obstetric indications for cesarean section should be determined individually for each pregnant woman in a multidisciplinary team, since orthopedic conditions may overlap with obstetric pathology in the pelvis.

## 1. Introduction

For several years, there has been an increasing number of cesarean sections performed. This fact is related to the expansion of obstetric indications for this procedure both on the part of the mother and the fetus [1]. The growing trend toward cesarean sections prompts analyses of the safety of this procedure in both women and their babies. Despite the advances that have been made in the fields of obstetrics and anesthesiology, cesarean section remains a procedure with a higher risk of intraoperative complications.

At the same time, the increasing proportion of people leading active lives and participating in sports is associated with an increased incidence of orthopedic injuries, including among women of reproductive age, with implications for their future pregnancies. The lack of clear recommendations prompts obstetricians to terminate pregnancies in women with a history of orthopedic conditions, most often by cesarean section [2]. Orthopedic indications for cesarean section are mostly related to diseases of the spine and hip joints that prevent full lower limb visitation at the hip joints during natural childbirth, as well as conditions after pelvic trauma and some cases of diastasis pubis. The lack of recommendations for the medical management of pregnant women with a history of orthopedic conditions and the limited experience of obstetricians in this area often pose a problem for decision-making, especially since pregnant women often do not have up-to-date imaging studies during pregnancy.

### Physiological Adaptation of the Musculoskeletal System during Pregnancy

**Figure 1:**
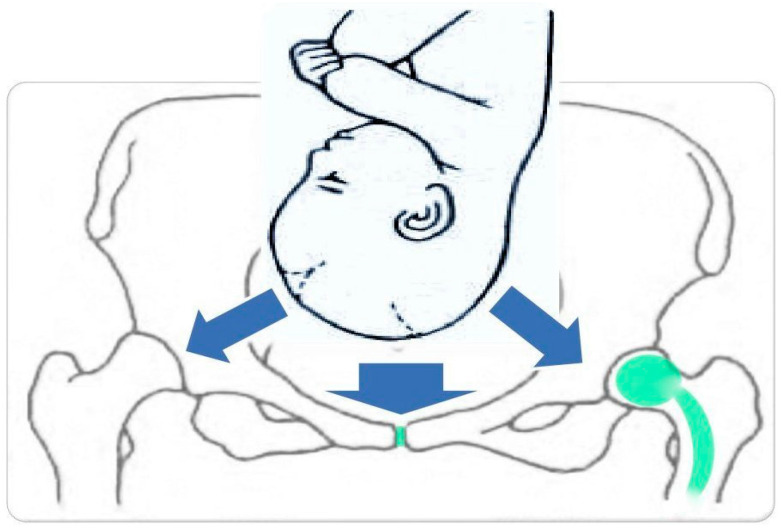
Forces acting on the various elements of the bony pelvis during childbirth (blue arrows). In green are marked sensitive areas: pubic symphysis and hip prosthesis, exposed to the action of strong forces, especially when abnormal mechanism of labor occurs.

During pregnancy, a number of changes occur in both biomechanical, hormonal and metabolic aspects, which promote an increased susceptibility to injury and symptomatic musculoskeletal dysfunction primarily in the musculoskeletal system. Hormonal changes during pregnancy, characterized by increases in relaxin, estrogen and progesterone levels, are potentially associated with ligamentous hyperelasticity and joint instability [3]. Under the influence of progesterone and relaxin during pregnancy, joint flaccidity begins to increase around the 10–12th week of pregnancy, reaching a maximum at or near the time of delivery [4]. The ligament laxity during pregnancy reaches its maximum at the second trimester [5]. In the first half of pregnancy, due to the predominance of anabolic responses, a significant weight gain is observed. It is the main cause of adaptive changes in the spine and pelvic rim bones. Weight gain accumulated mainly in the lower body and pelvic region is the main factor causing mechanical changes in the body during pregnancy in the form of pelvic anterior tilt, hip flexion contractures and lumbar hyperlordosis. The aforementioned changes are designed to compensate for the body’s imbalance, formed by the forward shift of the pregnant uterus and the growth of mammary glands, causing the woman to assume a very upright pose. Neuromuscular adaptations during pregnancy are also associated with an increase in the activation of the lumbo-pelvic muscles and a decrease in the strength of the pelvic floor muscles [3]. They are also the cause of the development of lumbopelvic pain. Perinatal adaptation also includes increased mobility of the tailbone, which tilts back under the pressure of the descending fetal head, increasing the circumference of the pelvic outlet (Figure 1).

During the perinatal period, changes in pelvic alignment are observed continuously and do not subside within the first month after delivery [6]. The risk of hip necrosis occurs at the end of pregnancy and after delivery, and appears to be decreasing rapidly within 9 months after childbirth [7]. Risk periods based on the timing of reported osteonecrosis and pregnancy confirmed the high incidence of osteonecrosis during pregnancy and the postpartum period, which was 71.8% compared with 28.2% during the antenatal period [7]. Although hip necrosis is a multifactorial disease, there are certain factors that predispose to it, such as lack of exercise in childhood and immobility in adulthood and dental problems as well [8]. In contrast, women diagnosed with transient hip osteoporosis had advanced maternal age, low body mass index (BMI), a family history of osteoporosis, a history of smoking and in vitro fertilization (IVF) pregnancies [9,10]. In those cases where hip movements are usually limited by pain, they often become non-obstetric indications for cesarean section [11]. In women undergoing cesarean section, more abdominal muscle exercises are needed to maintain pelvic stability [12].

## 2. Methods

This study is based on an analysis of available studies and articles, which focused on pregnancy and the most common orthopedic diseases. Given the extremely rapid advances in the surgical techniques used in orthopedics as well as the use of increasingly modern implant materials and the changing recommendations of scientific societies, available sources from the last 20 years were included in the analysis.

In this manuscript, a systematic research was conducted and Preferred Reporting Items for Systematic Reviews (PRISMA) guidelines were followed. The literature was searched using 5 databases (Cochrane Library, PubMed, Medline, Embase, Science Direct) from inception. The search strategy used a combination of keywords and Medical Subject Headings (MeSH). The MeSH terms used in the search strategy were as follows: “pregnancy”, “delivery”, “caesarean” in combination with “hip”, “pubic symphysis”, “pelvic bone fracture”. All articles collected through the e-search process used in this article were screened and reviewed by at least two authors.

### 2.1. Inclusion and Exclusion Criteria and Identification of Studies

**Figure 2:**
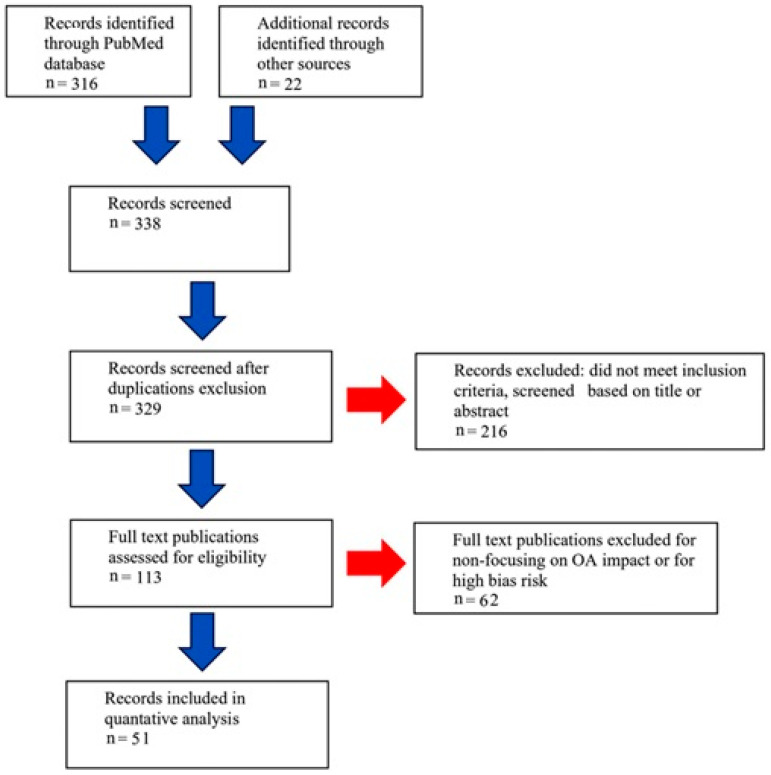
Flow diagram of literature search and selection process.

All studies that evaluated orthopedic interventions before or during pregnancy were eligible for inclusion (n = 338). The meeting abstracts or conference proceedings, articles duplicated in databases, articles unrelated to the main topic, conference reports, articles written in a language other than English and records which did not meet inclusion criteria were excluded from the analysis (n = 216). Finally, full-text publications evaluated for eligibility were included in the analysis (n = 113). Of the above, those publications that did not focus on open access (OA) impact or had high bias risk were excluded (n = 63). The availability of OA remains an important instrument for internal scientific communication and provides an opportunity to stay abreast of all developments in the field, especially for researchers from different regions and institutions. OA articles tend to have greater scientific and social impact than non-OA articles in the long term. Only current, full-text studies on the association between pregnancy and the most common orthopedic diseases were included in our review (n = 50). In the case of duplication of information in publications, those that contribute most to the topic under study were selected. The literature search and selection scheme is shown below (Figure 2).

### 2.2. Data Extraction

Due to the lack of available cohort studies and meta-analyses involving a large group of pregnant women, the high heterogeneity of studies and their results, a narrative synthesis of the conclusions of the included studies was conducted.

## 3. Results

A systematic search in Medline, Embase, PubMed, Science Direct and The Cochrane Library identified 39 articles directly related to the described four orthopedic-disorder-complicated pregnancies. Of these, 15 articles were related to postpartum pubic symphysis diastasis in pregnancy, 11 articles were related to lumbar disc herniation, six articles were related to a history of hip arthroplasty in pregnant women, and seven articles were related to a history of pelvic bone fracture in pregnant women. The age of the women in the included studies ranged from 18 to 37 years, with a mean of 29.15 (SD ± 4.35). The gestational age of the patients ranged from 37 to 41 weeks with a mean of 38w4d (SD ± 0.91).

## 4. Postpartum Pubic Symphysis Diastasis (PPSD)

The pubic symphysis (PS) is a cartilaginous joint that connects the pubic bones and encloses the anterior arch of the pelvic rim. It plays an important role in gait dynamics. Perinatal dissection of the PS is defined as pathological excessive separation of the pubic symphysis. Its incidence in varying degrees of severity ranges from 1/30,000 to 1/300 pregnancies [13]. Under physiological conditions outside of pregnancy, the width of the PS is about 4 to 5 mm, but during pregnancy, due to the relaxing effect of hormones, it increases by at least 2 to 3 mm. Normalization of PS dilation in pregnancy to 4–5 mm can occur by the 6th week after delivery [14]. Little is known about risk factors for PPSD. In a Sung JH et al. case–control study, the authors analyzed the cases of 21,131 women who gave birth by natural route, of whom 33 were diagnosed with symptomatic post-partum pubic diastasis. The incidence of PPSD was 0.156% (33/21,131) [13]. In this study, pre-pregnancy body mass index, weight gain during pregnancy, gestational diabetes, induction of labor, duration of labor, epidural anesthesia, vacuum-assisted delivery, episiotomy, neonatal sex and birth weight did not show to rise the incidence of PPSD. However, nulliparity was seen to be the only significant risk factor for PPSD. In most of these cases, conservative treatment such as bed rest, the use of analgesic drugs and belts placed on the women’s pelvis have been used successfully as effective treatment. A database analysis by Urraca-Gesto MA et al. found only 18 manuscript entries, of which 14 were case reports and four were case series. Consequently, the level of evidence for most of the selected studies was low [15]. Most of these studies conducted used bed rest as the main method of treating PPSD in the initial symptomatic period, mainly in the lateral recumbent position. Regardless of the form of treatment, the pain remained present in varying degrees of severity for 3 months postpartum, and complete resolution and elimination of the pain did not occur until 6 months postpartum. For conservative treatment, additional physiotherapy is recommended, including strengthening and stabilizing exercises. Among 4151 women who delivered 4554 babies analyzed by Yoo, 21 women were consulted in the orthopedic department for perinatal PS pain, and only 11 women were diagnosed with perinatal pubic symphysis diastasis [16]. Of these women, most required conservative treatment while two women underwent orthopedic surgery. However, the lack of detailed information from multiple studies precludes any recommendations on the best physiotherapy program for treating PPSD. Widening of the PS greater than 10 mm is a pathological finding [17]. If excessive dilation of the PS of more than 10 mm occurs, there is an inflammatory reaction accompanied by swelling of the soft tissues of the conjunctiva. Diastasis wider than 15 mm is considered a subdislocation and is usually associated with pain, swelling and sometimes deformity. Most cases can be treated conservatively. Sometimes, however, internal or external surgical stabilization may be required [18]. Macrosomia of the fetus, narrow pelvic dimensions, vaginal operative delivery and also previous pelvic trauma contribute to the above disorders in the perinatal period, but the above data are mainly based on studies conducted in small groups of patients [18]. Intrapartum risk factors for PPSD also include prolonged first period of labor and short second period of labor, dynamic uterine contractions with short intervals, epidural anesthesia, shoulder dystocia, forceps delivery and developmental dysplasia of the hip [19,20]. The use of the McRoberts maneuver in shoulder dystocia is also associated with a higher risk of pubic conjunctival dissection [21,22]. An inverse correlation was found between maternal age and the degree of dilatation of the PS in non-pregnant women. In contrast, this relationship was not observed in multiparous women [23]. Although it is a rare complication, it can lead to a number of symptoms and dysfunctions. Symptoms of diastasis pubis usually appear at the 36–38th week of gestation. Characteristic of the condition at that time is pain and difficulty walking and urination disorders. There is also a feeling of instability of the pelvis, hips and lower lumbar spine, a positive Trendelenburg sign and a waddling gait. In severe cases, hematomas, pelvic fractures, damage to the sacroiliac joint and damage to the urinary tract can occur [24]. However, the severity of the symptoms does not always correlate with the degree of divergence of the pubic conjunctiva. Sometimes, pelvic pain can result from chronic instability of the anterior pelvic ring. The pain in this case is usually localized to the suprapelvic region or inner thigh, is often associated with lower back or buttock pain, and can be aggravated by activity, direct impact or pressure on the pelvic ring [25]. Imaging studies such as radiography, ultrasound and MRI are diagnostic methods that help confirm the PPSD diagnosis [26].

PPSD does not pose a significant risk to the pregnancy and is a relative indication for its termination by cesarean section. In most women with an asymptomatic or clinically uneventful course of it, vaginal delivery is recommended, even when its width is about 50 mm. In a study by Rustamova S. et al., a significant increase in the width of the conjunctiva was observed between the first and second periods of labor at the two extremes of measurement [23]. Widening was observed in 94% at the highest and 59% at the narrowest width of the PS. Natural childbirth can exacerbate local symptoms of pubic symphysis diastasis and cause them to persist longer after delivery. Caesarean section (CS) is indicated when painful restriction of lumbar spine mobility and hip joint abduction prevent normal labor action. The decision to perform CS is made by the obstetrician after taking into account the written opinion of the orthopedist, exceptionally in a separation of less than 10 mm, more often 10–20 mm, and most often more than 40 mm. Patients with a PPSD of 40 mm can be treated nonoperatively, while patients with larger pubic conjunctival dissection with concomitant increased clinical symptoms often undergo surgical stabilization. In patients with previous pubic symphysis diastasis during a previous vaginal delivery, given the significant risk of repeated separation of the pubic conjunctiva and recurrence of symptoms, a repeat cesarean section is recommended [22]. However, performing a prophylactic cesarean section to prevent PPSD does not prevent physiologic separation of the PS [14]. Given the lack of standards for the criteria for diagnosing PPSD and the occurrence of PPSD mostly after delivery, based on the cited data, prophylactic cesarean section is not recommended for the prevention of PPSD. The results of the cited studies sometimes diverge which is due to the different way PPSD is imaged in different medical centers as well as, in the absence of clearly defined guidelines, the use of internal criteria for the evaluation of PPSD.

## 5. Lumbar Disc Herniation (LDH)

Lumbosacral pain during pregnancy, especially toward the end of pregnancy, is a common complaint, affecting about half of pregnant women. Although the presence of back pain during pregnancy is very common, the incidence of its occurrence secondary to lumbar disc herniation in pregnancy is low [27]. True disc herniation is extremely rare, occurring in 1 in 10,000 patients [28]. There are suggestions that low female parity may possibly be related to the development of spinal degeneration [29]. In contrast, the association of cauda equina syndrome as a result of a herniated disc during pregnancy is extremely rare [27]. Lumbar disc herniation has been linked to the inflammatory response that occurs, generating its symptoms [30]. Similar to pubic conjunctival distention, it has been suggested that relaxin release of the third trimester of pregnancy, may predispose to massive lumbar disc prolapse [31]. In most cases, radiculopathy caused by a lumbar disc herniation does not require surgery, and treatment is based on rehabilitation and pharmacology. A review by Paslaru F.G. et al. analyzed 30 studies involving 52 patients [32] in which conservative treatment was associated with better long-term therapeutic effects compared to surgical treatment [32]. Although pregnancy itself is not a contraindication to surgical treatment of LDH at any stage of its duration [33], the most frequently chosen treatment method is conservative treatment until delivery [34,35]. There are various conservative treatments that can provide relief from herniated disc pain during pregnancy such as physical therapy, acupuncture, heat and ice therapy, prenatal massage, transcutaneous electrical nerve stimulation (TENS) units. Progression to cauda equina syndrome or neurological deficit mostly need surgical intervention. Surgery during pregnancy should be reserved only for those women who have significantly pronounced clinical symptoms and this decision should be made by orthopedists. It is important to take special care and protect the fetus and also the position of the operation adapted to the weeks of gestation. Optimal surgical access with minimal blood loss by reducing epidural venous pressure is provided by the supine position, which is most commonly used during the first and early second trimester of pregnancy. In the third trimester, the supine position is difficult to achieve in the pregnant uterus. Importantly, pregnancy at any stage is no contraindication to magnetic resonance imaging scan, epidural and/or general anesthesia, and surgical disc excision. Using preoperative MR imaging and full-endoscopic interlaminar discectomy, minimally invasive spine surgery without X-rays is possible to perform in pregnant women with lumbar disc herniation. However, the decision on how to terminate pregnancy in women with symptomatic LDH or after LDH surgery is mostly made by the obstetrician and the recommended method of termination of pregnancy is cesarean section, performed to avoid worsening of symptoms and progression to cauda equina syndrome [32,36]. After the LDH surgery is performed during pregnancy, the cesarean section seems to be preferred compared to vaginal delivery to avoid worsening symptoms and progression to the cauda equina syndrome. In the study conducted by Brown M.D. et al., the authors showed that inducing labor in women with a lumbar hernia can result in increased neurologic damage due to the increased epidural venous pressure that occurs during pushing [37]. In some cases, there is a high likelihood of increased symptoms during pregnancy and delivery, e.g., prolapsed disc, vertebroplasty, grade III–IV with symptoms of static spinal insufficiency which, a priori, may be an indication for surgical termination of pregnancy. In women who have undergone disc excision surgery before pregnancy, it is recommended that the period from surgery to becoming pregnant be at least 6 months.

## 6. Status Post Hip Arthroplasty

About 2–3% of all total hip replacements are performed in women of childbearing age, where cementless prothesis implantations are the most common. In such cases, body weight increases by up to 0.5 kg. The additional burden during pregnancy with superimposed increased hip mobility during pregnancy and 3 months after delivery may carry a potential increased risk of endoprosthesis dislocation. According to the literature, hip dysplasia is not associated with high-risk complications during pregnancy or with increased difficulty in vaginal delivery [38]. Similarly, pregnancy after total hip arthroplasty (THA) is not associated with decreased endoprosthesis survival, according to Sierra R.J. et al. [39]. Childbirth does not appear to reduce the survival rate of total hip replacement implants from which it follows that women should not fear or avoid becoming pregnant after total hip replacement [40]. Having undergone hip endoprosthesis surgery is not a contraindication to pregnancy. On the other hand, pregnancy and childbirth in women after hip replacement do not pose an absolute threat to the implant and the condition after hip arthroplasty does not have a detrimental effect on subsequent pregnancies in terms of maternal or child health. There is also no increase in pregnancy complications or delivery difficulties resulting from hip replacement and, therefore, the pregnancy status does not appear to have a deleterious effect on THA [41]. Based on the literature analyzed in this work, the decision to perform a cesarean section is rarely ultimately influenced by hip dysplasia or previous surgery for hip dysplasia in a pregnant woman. Despite reports of the possibility of safe natural childbirth after THA and the lack of reported adverse effects on overall outcome, function and radiographic appearance after THA [42], some women and their obstetricians are more likely to choose elective cesarean section to terminate their pregnancies, despite the lack of scientific basis for such action. Childbirth after THA can take place without much restriction on body position, but care should be taken not to sit in too deep chairs or on very soft beds, not to exceed 90 degrees of joint flexion angle. On the other hand, the absolute indications for cesarean section in some women after hip replacement are mainly due to anatomical conditions that make it impossible to carry out a natural delivery. Considering the birth status of the newborns, women after THA have a higher risk of preterm delivery (aOR 3.58, *p* ≤ 0.001), giving birth to babies small for gestational age (aOR 2.83, *p* = 0.006) and with low birth weight (aOR 4.79, *p* ≤ 0.001) compared to the control group [43]. In the study by Kuitunen I. et al., attempted natural childbirth was more often terminated by emergency cesarean section in the group of women after THA compared to the controls [39 (28.9%) vs. 150 (11.6%), *p* ≤ 0.001]. Adverse pregnancy outcomes were also more common after THA compared to pregnancies before THA [43]. Taking into account the scientific reports included in this study, there are no absolute obstetric contraindications for women after THA to give birth vaginally.

## 7. Pelvic Bone Fracture

There has been an increasing rate of pelvic bone injuries in recent years. In a study by Lundin N. et al., the incidence increased from 64 to 80 per 100,000 person years between 2001 and 2016, the vast majority (74%) of whom were women [44]. Since some of the women in this group remain of reproductive age, pregnancy and childbirth pose a challenge not only for orthopedists but also for obstetricians. Treatment of fractures occurring in pregnant patients is particularly challenging, given the anatomical and physiological changes in pregnancy increase the complexity of treatment. Maternal trauma increases the risk of fetal loss, premature delivery, placental abruption, cesarean section and maternal death [45]. According to the literature, with highly specialized medical care in pregnant women after pelvic fracture, pregnancies and deliveries can be uncomplicated [46]. A lower cumulative birth rate (HR 0.79, CI 0.64–0.97) is observed among women with a pelvic fracture aged 25–34 years compared with controls [47]. There are extremely rare cases where a pelvic ring rupture has occurred during childbirth. The mechanism involves the hormonal relaxation of the pelvic ligaments in conjunction with the strong movement of the fetal head [48]. Although a history of pelvic bone fracture is not an absolute indication for cesarean section and vaginal delivery is possible, the rate of cesarean sections among these women is significantly increased, and more than half of the indications for surgical termination of pregnancy are due to patient and obstetrician preference [49]. In a systematic review conducted by John T. Riehl, out of 148 patients who underwent labor after pelvic fracture, 79 (53%) gave birth naturally and 69 (47%) underwent cesarean section [49]. Applied orthopedic treatment, minor pelvic deformity or postoperative bone fusions are not absolute indications for cesarean section. Physicians’ concerns about the possibility of successful natural childbirth make it necessary to develop guidelines and objective indications for attempting normal delivery after pelvic fracture [46]. A study by Vaajala M. et al. analyzed a total of 2878 women with pelvic fracture and 1330 women with hip fracture, taking into account the time elapsed from injury to pregnancy and delivery. Of these, 586 (20.4%) women gave birth within the next 14 years after the pelvic fracture and 147 (11.0%) women after the hip fracture [47]. Women with pelvic fracture had more frequent cesarean sections in each period analyzed. The aOR for CS was 1.62 (CI 1.22–2.12) in the first 5 years, 1.87 (CI 1.33–2.62) in years 5–10 and 1.97 (CI 1.11–3.41) in 10–14 after injury. Also, women with hip fractures had significantly higher odds of CS within the first 5 years after fracture (aOR 1.64, CI 1.40–2.67) [50]. The results of this study suggest that vaginal delivery is generally possible within a relatively short period of time after a hip or pelvic fracture. After a pelvic bone fracture, it is usual to perform a MR-pelvimetry to look at the shape of the pelvic bone and search for signs of reduced pelvic bone at the end of pregnancy (around 36–37 weeks of gestation). MR-pelvimetry is useful to explore the pelvic bone and to measure the anteroposterior diameter of the pelvic inlet (APDPI), transverse inlet diameter (TID) and bispinous diameter (BSD) through the ischial spines. In normal pregnancies without any orthopedic issues, it is used mostly when the babies are in breech presentation. If one of the measurements is reduced, this is a contraindication for vaginal delivery in case of breech presentation. In case of cephalic presentation, the bones of the baby’s skull have the time to adapt to the pelvic bone of the mother during labor [51].

## 8. Limitations and Bias

All included studies were either case reports or case series, which limited their methodological quality and increased the risk of bias. A particular difficulty was the lack of detailed descriptions and applied surgical techniques used in the analyzed patients, which may result in different functional effects of the musculoskeletal organ, and consequently its different adaptation during pregnancy. In some of the analyzed cases, at the time of labor, it was not possible to conduct an orthopedic consultation regarding the method of ending the pregnancy, which constituted an additional decision-making burden for the obstetrician and was therefore associated with an unobjective assessment of the chance of delivery and the risk of complications, consequently leading to an easier decision to perform cesarean section.

## 9. Conclusions

The increase in the percentage of cesarean sections performed, including for orthopedic indications, is a visible trend in obstetrics. The lack of precise recommendations on orthopedic indications for termination of pregnancy by cesarean section, the possibility of orthopedic consultations in obstetrics departments, as well as the fear of patients’ claims make doctors increasingly opt for prophylactic cesarean sections. Orthopedic indications for cesarean section in many cases should not be treated imperatively, since natural delivery after correct fusion of a pelvic fracture, implantation of a hip endoprosthesis or a limited dissection of the pubic symphysis is possible and is not associated with a higher risk of obstetric or orthopedic complications. It should be noted that the cases of LDH with neurosensory deficiency are an indication for emergency spinal surgery in any stage of pregnancy. Extra-obstetric indications for cesarean section should be determined individually for each pregnant woman in a multidisciplinary team, since orthopedic conditions may overlap with obstetric pathology in the pelvis.

## Data Availability

Not applicable.
